# Supplementary material for: Correlation of Clinical Parameters with Intracranial Outcome in Non-Small Cell Lung Cancer Patients with Brain Metastases Treated with Pd-1/Pd-L1 Inhibitors as Monotherapy
Source: Cancers (Basel). 2021 Mar 29;13(7):1562. doi: 10.3390/cancers13071562 (PMC8036699; doi:10.3390/cancers13071562)
Supplement: Supplementary file 1 [file cancers-13-01562-s001.pdf]

cancers

Article

Correlation of Clinical Parameters with Intracranial Outcome in Non-Small Cell Lung Cancer Patients with Brain Metastases Treated with Pd-1/Pd-L1 Inhibitors as Monotherapy

Konstantinos Rounis [1,2,3,*], Marcus Skribek [1,2], Dimitrios Makrakis [3,4], Luigi De Petris [1,2], Sofia Agelaki [3], Simon Ekman [1,2,†] and Georgios Tsakonas [1,2,†]

1 Thoracic Oncology Center, Theme Cancer, Karolinska University Hospital, 17164 Stockholm, Sweden; marcus.skribek@ki.se (M.S.); luigi.depetris@ki.se (L.D.P.); simon.ekman@ki.se (S.E.); georgios.tsakonas@ki.se (G.T.)
2 Department of Oncology-Pathology, Karolinska Institutet, 17177 Stockholm, Sweden
3 Department of Medical Oncology, University General Hospital, 71110 Heraklion, Crete, Greece; dmakrak@uw.edu (D.M.); agelaki@uoc.gr (S.A.)
4 Division of Oncology, University of Washington Medical School, Seattle, WA 98195, USA
* Correspondence: kostas@rounis.gr; Tel.: +306976592930
† Equal contribution.

Citation: Rounis, K.; Skribek, M.; Makrakis, D.; De Petris, L.; Agelaki, S.; Ekman, S.; Tsakonas, G Correlation of Clinical Parameters with Intracranial Outcome in Non-Small Cell Lung Cancer Patients with Brain Metastases Treated with Pd-1/Pd-L1 Inhibitors as Monotherapy. *Cancers* **2021**, *13*, x. https://doi.org/10.3390/xxxxx

Academic Editor: Johan Max Kros

Received: 16 February 2021
Accepted: 24 March 2021
Published: date

Publisher's Note: MDPI stays neutral with regard to jurisdictional claims in published maps and institutional affiliations.

Copyright: © 2021 by the authors. Licensee MDPI, Basel, Switzerland. This article is an open access article distributed under the terms and conditions of the Creative Commons Attribution (CC BY) license (http://creativecommons.org/licenses/by/4.0/).

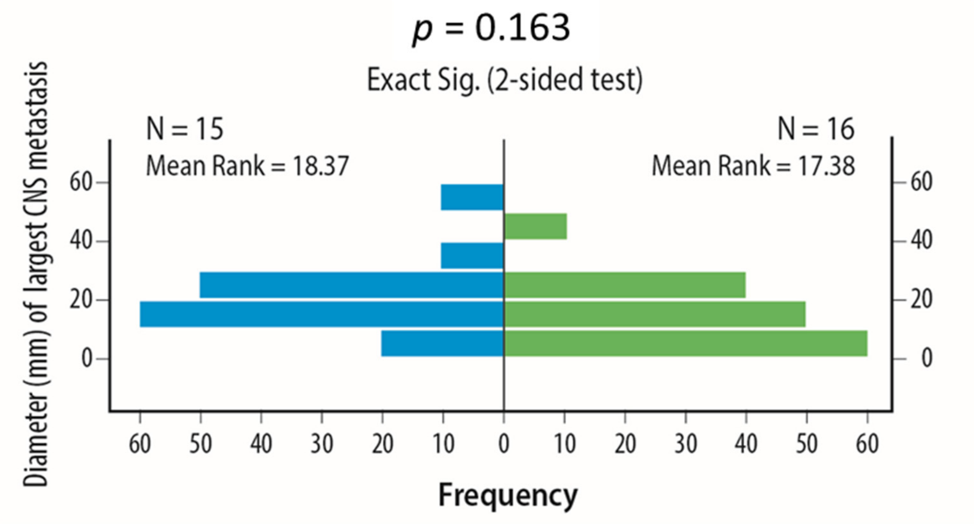

Figure S1. Effect of the diameter of the largest CNS metastases on IC DC rates (Mann-Whitney U test, CI 95%). On the left side is the mean diameter of the largest CNS metastases of the patients that experienced intracranial disease control and on the right side the mean diameter of the largest CNS metastases of those who developed CNS disease progression after ICI administration.

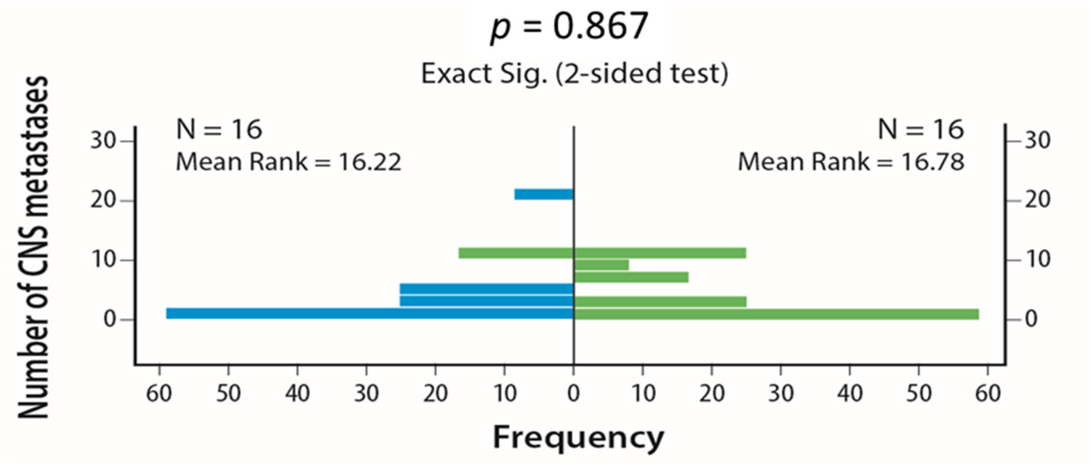

Figure S2. Effect of number of CNS metastases on IC DS rates (Mann-Whitney U test, CI 95%). On the left side is the mean number of CNS metastases of the patients that experienced intracranial disease stabilization and on the right side the mean number of CNS metastases of those who developed intracranial progressive disease after ICI administration.

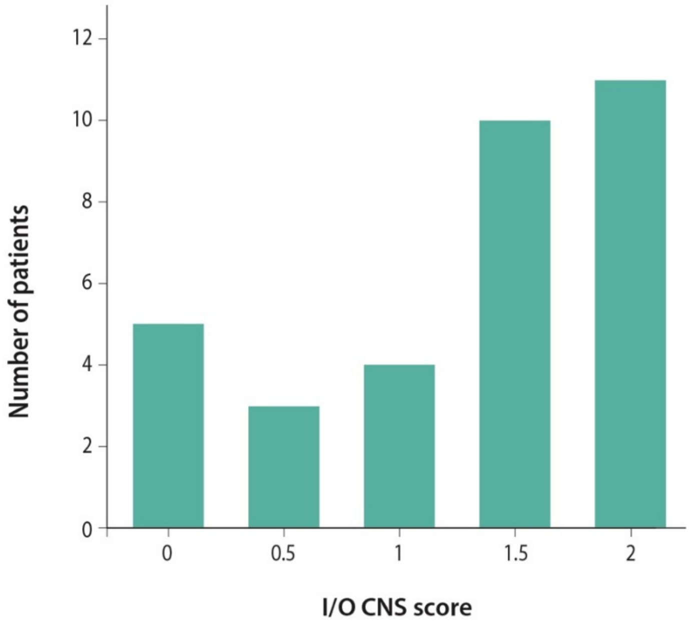

Figure S3. Bar plot depicting the distribution of I/O CNS score amongst the patients of our cohort.

Table S1. Chi-square test examining the effect of the studied parameters on intracranial disease control (PR or SD) as result of ICI administration (i stands for intracranial).

Parameter	Total *n*=33	PRi+SDi	PDi	Pearson Chi-square Test (1-df) (95% CI)
Age				
<70 years old	20	13	7	*p*=0.019
>70 years old	13	3	10	
Gender				
Male	16	9	7	*p*=0.387
Female	17	7	10	
Performance status				
0-1	25	14	11	*p*=0.127
2	8	2	6	
Histology				

Adenocarcinoma	29	16	13	p=0.038
Non-adenocarcinoma	4	0	4	
PDL1 levels in extracranial lesions				
<50%	12	9	3	p=0.018
≥50%	14	4	10	
Disease burden				
1-2 organs with metastases	13	5	8	p=0.353
>2 organs with metastases	20	11	9	
Primary CNS metastases at diagnosis				
Yes	24	15	9	p=0.011
No	9	1	8	
Number of CNS metastases				
≤3	21	10	11	p=0.895
>3	12	6	6	
Size of largest CNS metastasis				
≤20mm	19	8	11	p=0.379
>20mm	12	7	5	
Neurological symptoms due to CNS metastases				
Yes	18	10	8	p=0.373
No	15	6	9	
Previous RT for CNS metastases				
Yes	18	12	6	p=0.022
No	15	4	11	
CNS response to previous systemic treatment*				
PRi+SDi	7	7	0	p=0.002
PDi	21	7	14	
Active BM				
Yes	25	8	17	p=0.001
No	8	8	0	
Line of treatment				
1st	5	1	4	p=0.166
2nd or subsequent	28	15	13	
Steroids use > 10 mg for ≥ 10 days				
Yes	16	6	10	p=0.221
No	17	10	7	
